# Supplementary material for: The landscape of myeloid and astrocyte phenotypes in acute multiple sclerosis lesions
Source: Acta Neuropathol Commun. 2019 Aug 12;7:130. doi: 10.1186/s40478-019-0779-2 (PMC6689891; doi:10.1186/s40478-019-0779-2)
Supplement: Supplementary file 1 — Supplementary tables and figures referred to in the article text. (DOCX 16375 kb) [file 40478_2019_779_MOESM1_ESM.docx]

**ADDITIONAL FILE 1**

**The landscape of myeloid and astrocyte phenotypes in acute multiple sclerosis lesions**

Calvin Park^1^, Gerald Ponath^1^, Maya Levine-Ritterman^1^, Edward Bull^1^, Eric C. Swanson^2^, Philip L. De Jager^3^, Benjamin M. Segal^4^, David Pitt^1^

*^1^ Department of Neurology, Yale School of Medicine, New Haven, CT, USA*

*^2^Fluidigm Corporation, Markham, ON, Canada*

*^3^Department of Neurology, Columbia University Medical Center, New York, NY*

*^4^Department of Neurology, University of Michigan, Ann Arbor, MI, USA*

Corresponding author: David Pitt, Email: [david.pitt@yale.edu](mailto:david.pitt@yale.edu)

**CONTENTS**

**Supplementary Tables Page**

Table S1 (Antibodies used for brightfield and fluorescence histology) 2

Table S2 (Metal-conjugated antibodies used for IMC) 3

**Supplementary Figures**

Figure S1 4

Figure S2 5

Figure S3 6

Figure S4 7

Figure S5 8

Figure S6 9

Figure S7 10

Figure S8 11

Figure S9 12

| **Primary Antibody** | **Host** | **Clone** | **Company** | **Target/Significance** | **Primary Antibody Dilution** | **Secondary Antibody** | **Secondary Antibody Dilution** |
| --- | --- | --- | --- | --- | --- | --- | --- |
| MBP | Rat | 12 | Millipore-Sigma | Myelin Basic Protein | 1:500  (Brightfield) | Invitrogen, 629540 | 1:500 (Brightfield) |
| CD68 | Rabbit | D4B9C | Cell Signaling | Activated macrophage/microglia transmembrane glycoprotein | 1:500 (Brightfield)  1:100  (Fluorescence) | Vector Laboratories, BA-1000 (Brightfield)  Invitrogen, A32731  (Fluorescence) | 1:500 (Brightfield)  1:1000  (Fluorescence) |
| MAP2 | Mouse | 5H11 | Novus | Neuron structural protein | 1:200  (Brightfield) | Vector Laboratories, BA-9200 | 1:500 (Brightfield) |
| CD74 | Mouse | LN2 | BioLegend | Microglial subtype marker | 1:100  (Fluorescence) | Invitrogen, B40961 | 1:3 (Tyramide Kit Fluorescence) |

**Additional file 1: Table S1** Antibodies used for brightfield and fluorescence histology

| **Antibody** | **Clone** | **Company** | **Target/Significance** | **Conjugated Metal Isotope** | **Conjugation Source** | **Dilution** |
| --- | --- | --- | --- | --- | --- | --- |
| LAMP1/CD107a | H4A3 | Fluidigm | Lysosomes | 151Eu | Fluidigm | 1:100 |
| CD3 | Polyclonal | Fluidigm | T cells | 170Er | Fluidigm | 1:75 |
| CD45 | 2B11 | Fluidigm | Leukocyte activation | 152Sm | Fluidigm | 1:100 |
| CD68 | KP1 | Fluidigm | Activated macrophages/microglia | 159Tb | Fluidigm | 1:300 |
| CD86 | Polyclonal | R&D | “M1” co-stimulatory molecule | 171Yb | Our Lab | 1:100 |
| Ferritin heavy chain | 1-2.3.1.2 | Millipore-Sigma | Iron storage | 158Gd | Our Lab | 1:400 |
| HLA-DR | YE2/36 HLK | Fluidigm | MHC Class II | 174Yb | Fluidigm | 1:200 |
| Mac2 | M3/38 | Fluidigm | “M2” activation | 153Eu | Fluidigm | 1:600 |
| MerTK | y323 | abcam | “M2” phagocytosis | 148Nd | Our Lab | 1:50 |
| PLP | plpc1 | Bio-Rad | Myelin | 142Nd | Our Lab | 1:100 |
| S100B | S100B/1706R | Novus | Astrocytes | 167Er | Our Lab | 1:400 |
| TIM-3 | D5D5R | Fluidigm | Co-inhibitory molecule | 154Sm | Fluidigm | 1:100 |
| Vimentin | RV202 | Fluidigm | Hypertrophic astrocytes | 143Nd | Fluidigm | 1:100 |

**Additional file 1: Table S2** Metal-conjugated antibodies used for IMC

**
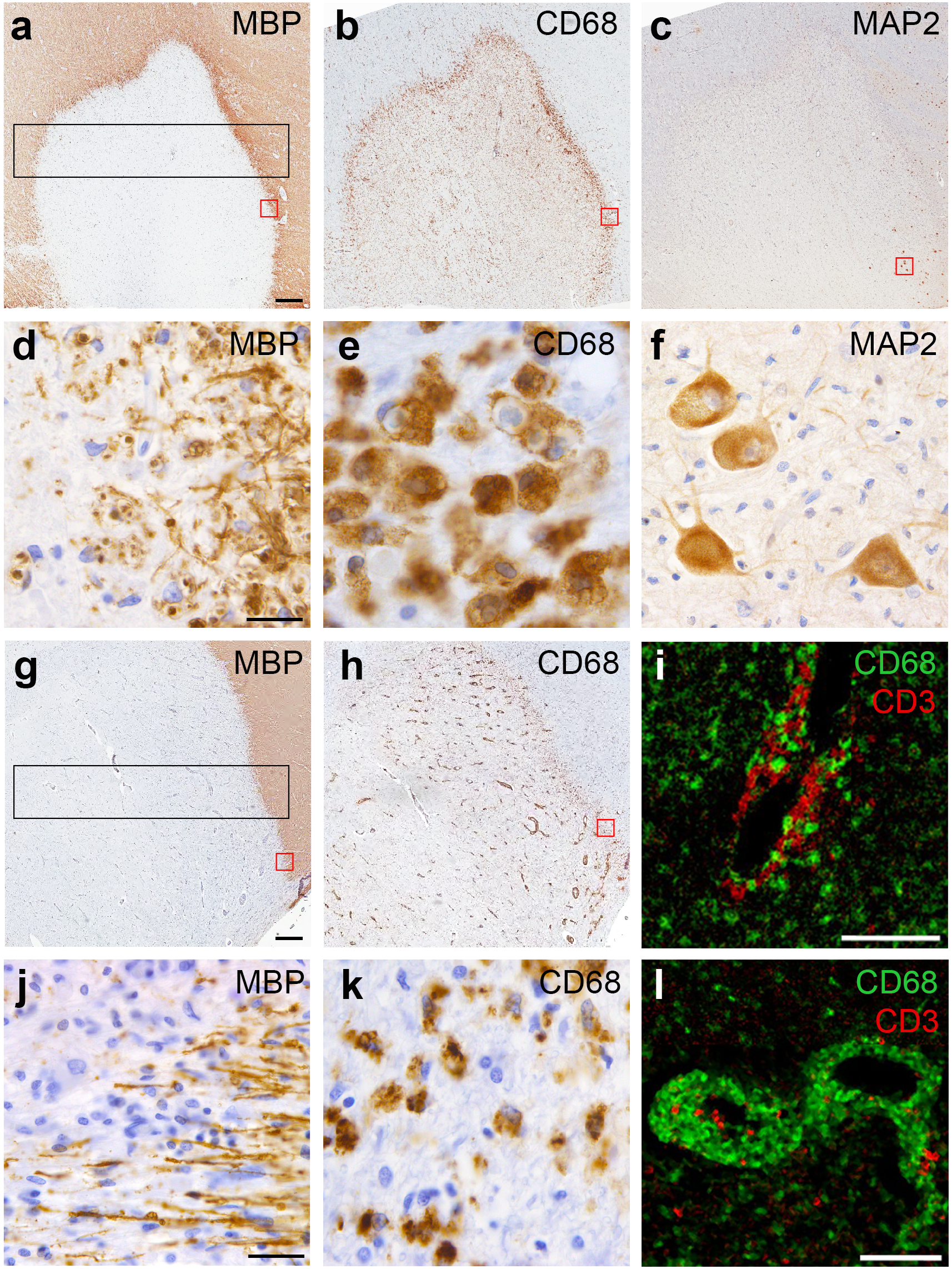
**

**Additional file 1: Figure S1** Brightfield and imaging mass cytometry images of the MS lesions examined in this study. **(a-f)** Brightfield staining of the early, actively demyelinating lesion for **(a)** myelin basic protein (MBP), **(b)** CD68 (macrophages/microglia) and **(c)** the neuronal marker MAP2. The black rectangle in **(a)** shows the region that was laser-ablated for IMC, capturing the NAWM, hypercellular rim on both sides and lesion center. Red boxes in **a-c** correspond to magnified rim areas in **d-f,** demonstrating in **(d)** condensed myelin fragments, indicative of ongoing demyelination, **(e)** dense infiltration with foamy macrophages and **(f)** dispersed neuronal cell bodies in the right lesion rim. **(i)** Merged IMC images of perivascular CD68^+^ (green) and CD3^+^ (red) cells in the early lesion.

**(g, h, j, k)** Brightfield staining against MBP and CD68 in the late, post-demyelinating lesion. The black rectangle in **(g)** shows the region laser-ablated for IMC. **(j, k)** Details from **(g)** and **(h)** showing absence of myelin fragments and reduced myeloid cell density at the lesion rim compared to **(e)**. **(l)** Merged IMC images of perivascular CD68^+^ (green) and CD3^+^ (red) cells in the late lesion. Brightfield images show hematoxylin counterstaining. Scale bars **a-c, g, h** = 500 μm. Scale bars **d-f, j, k** = 25 μm. Scale bars **i, l** = 100 μm

**
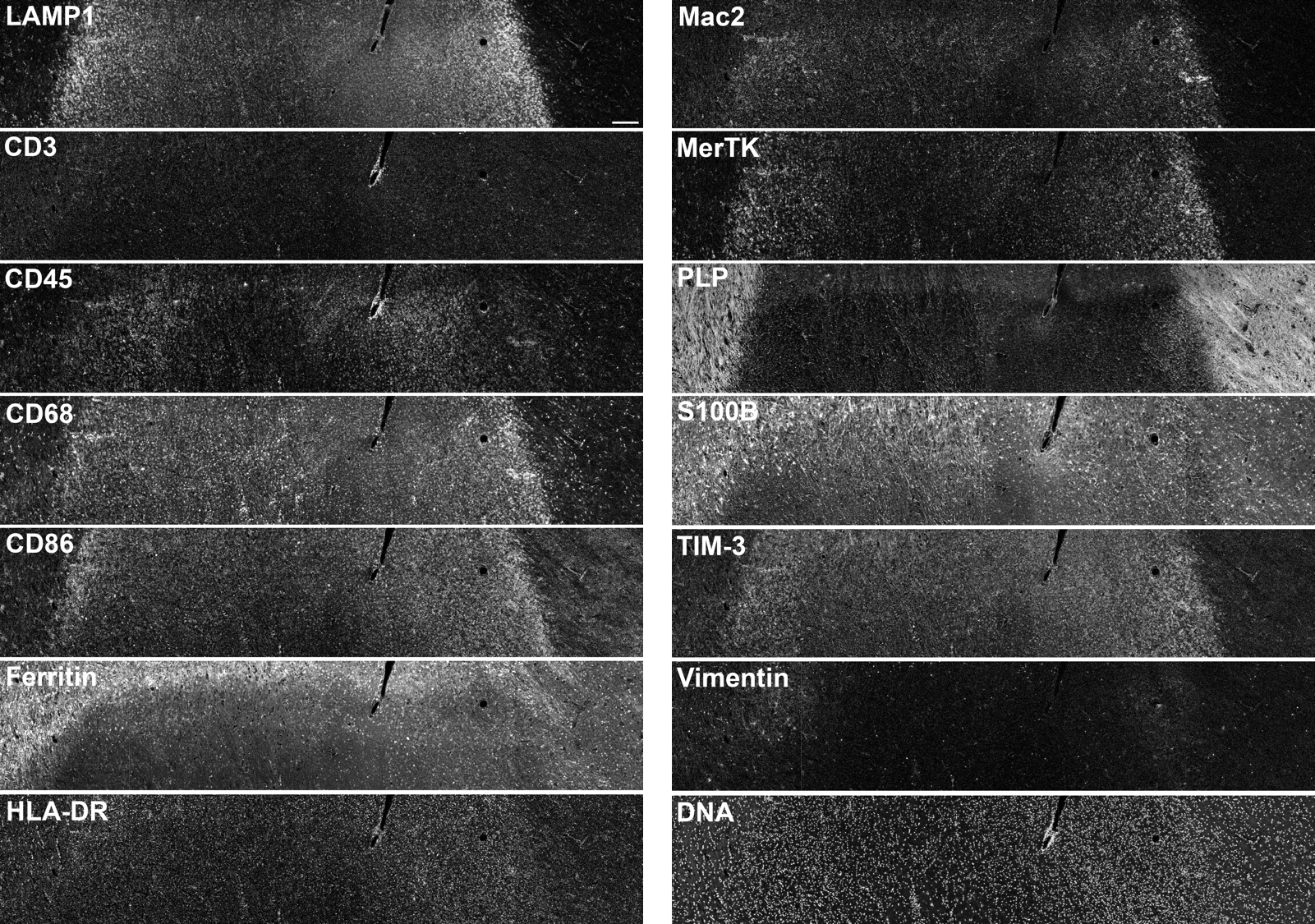
**

**Additional file 1: Figure S2** Imaging mass cytometry histology images of the early lesion. The thirteen markers used for cell clustering as well as DNA counterstaining are shown. Scale bar = 200 μm

**
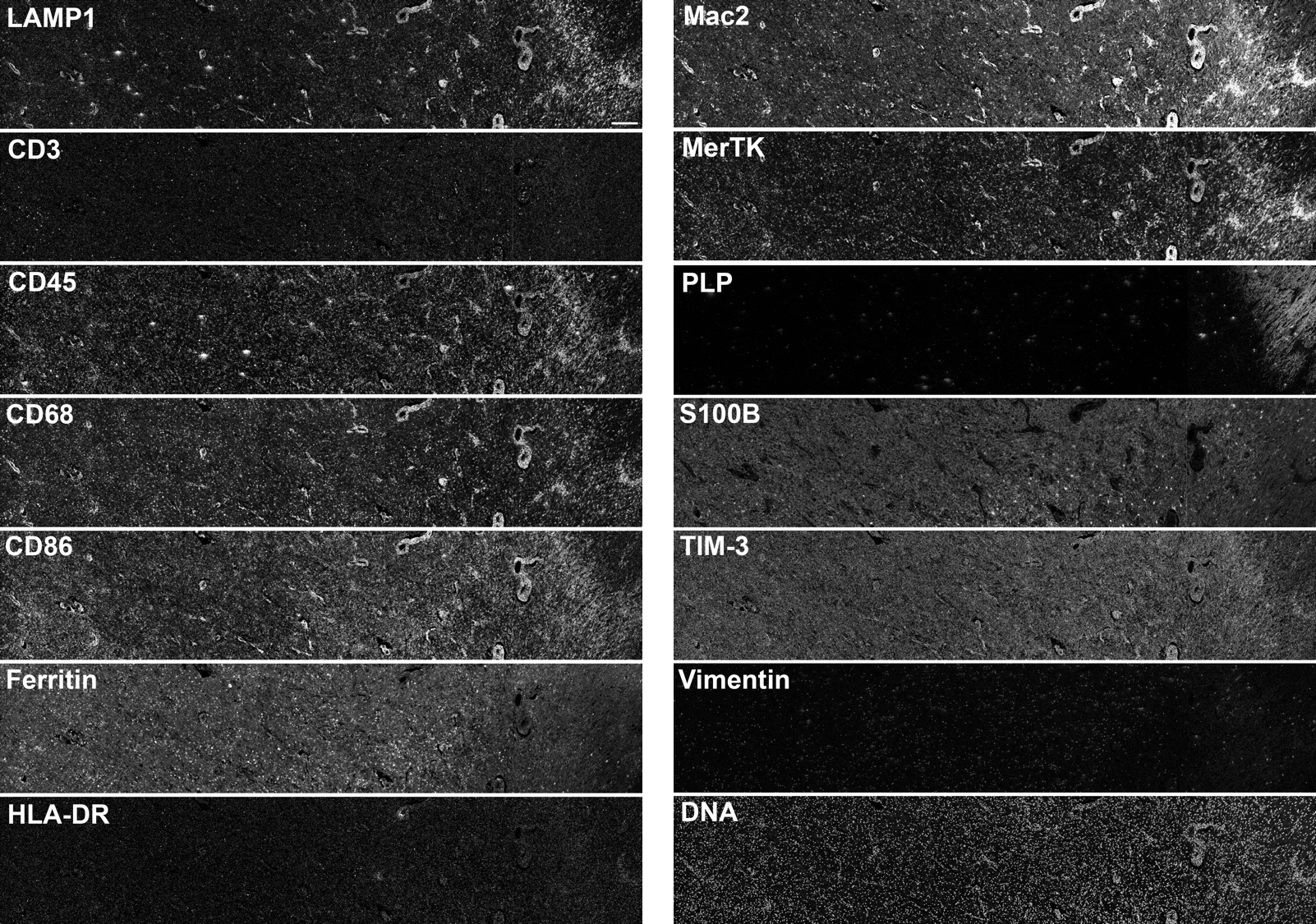
**

**Additional file 1: Figure S3** Imaging mass cytometry histology images of the late lesion. The thirteen markers used for cell clustering as well as DNA counterstaining are shown. Scale bar = 200 μm

**
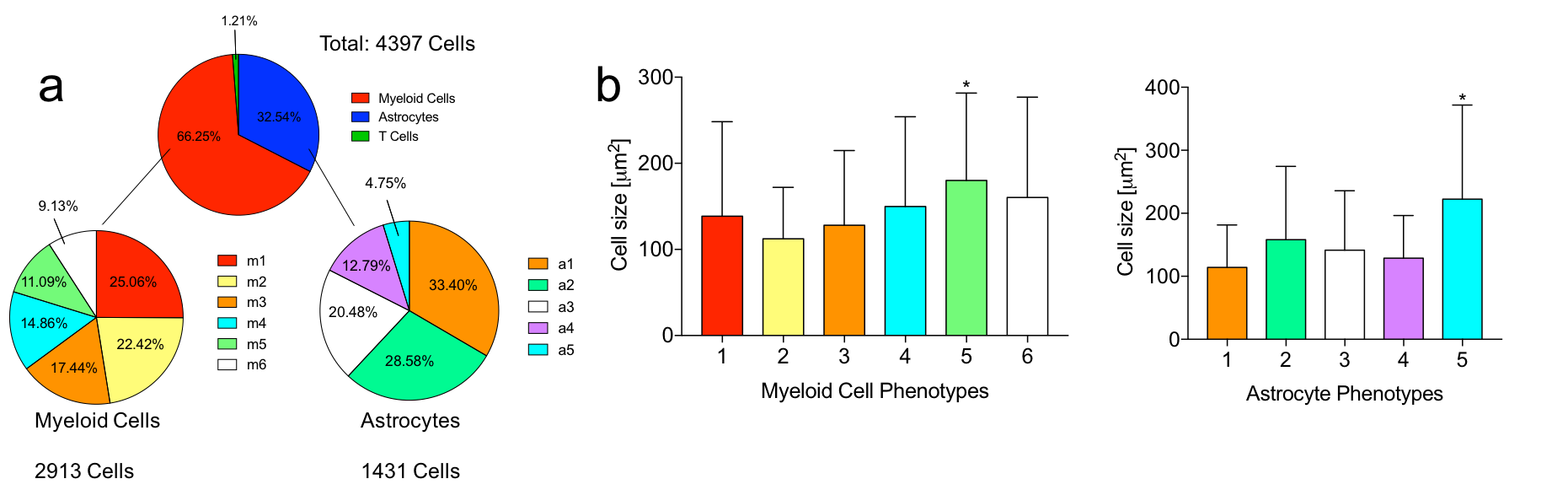
**

**Additional file 1: Figure S4** Early lesion cell phenotype frequency and size. **(a)** Relative quantities of each cell phenotype. **(b)** Cell phenotype size. Cells of myeloid phenotype 5 (m5), which occupy the white matter outer rim, are significantly larger than all other myeloid phenotypes. Similarly, cells of astrocyte phenotype 5 (a5), which occupy the white matter outer rim, are significantly larger than all other astrocyte phenotypes. Data represent means + standard deviation. *p < 0.0001 by one-way ANOVA followed by the Tukey-Kramer multiple comparison test

**
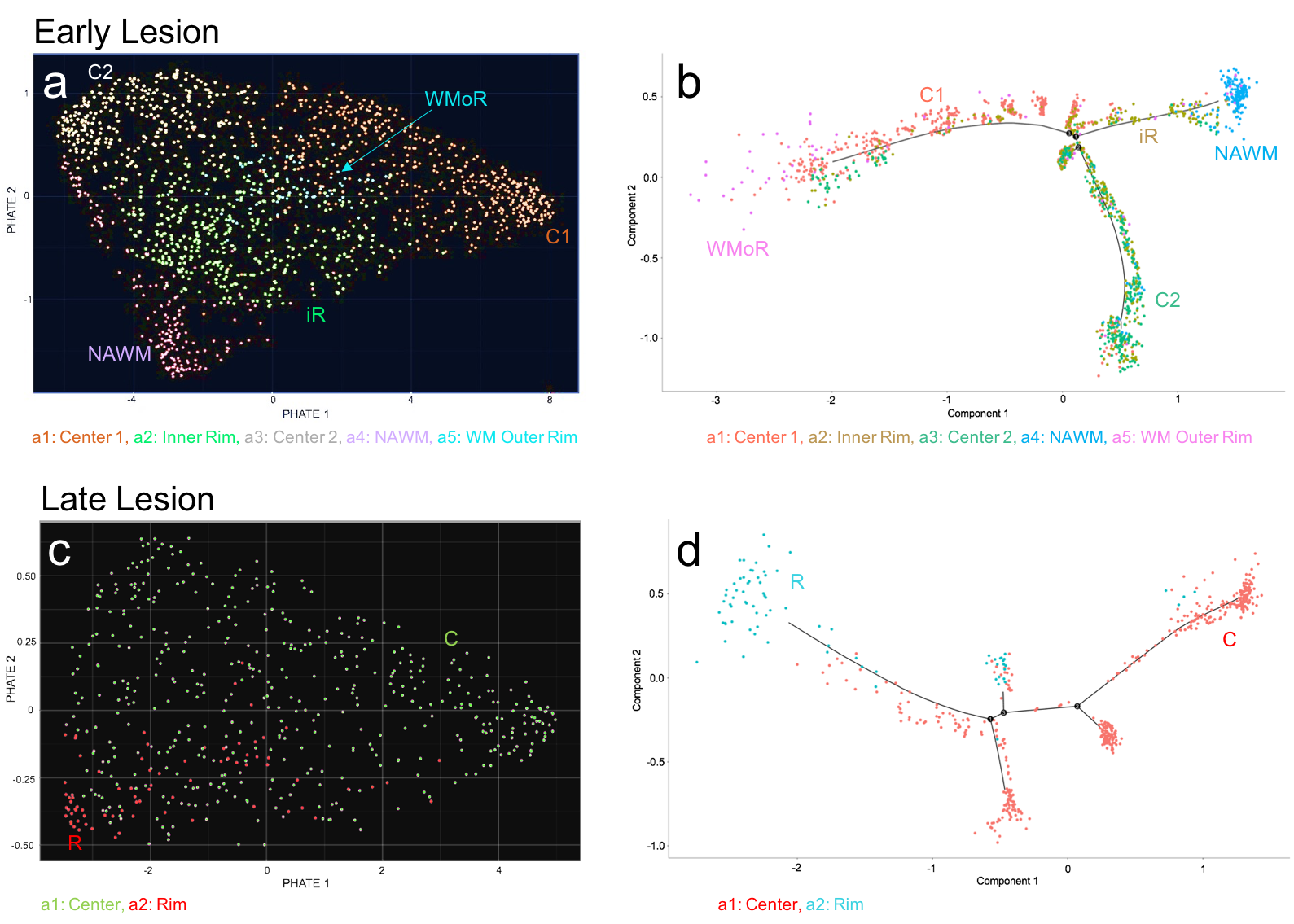
**

**Additional file 1: Figure S5** Analyses of astrocyte phenotype transitions in the **(a, b)** early and **(c, d)** late lesion. Both PHATE mapping **(a, c)** and Pseudotime analyses **(b, d)** demonstrate no linear phenotype transition in either lesion. Phenotype color schemes on the PHATE and Pseudotime plots reflect the color palettes specific to each analysis. R = rim; WMoR = white matter outer rim; iR = inner rim; C = center; NAWM = normal-appearing white matter

**
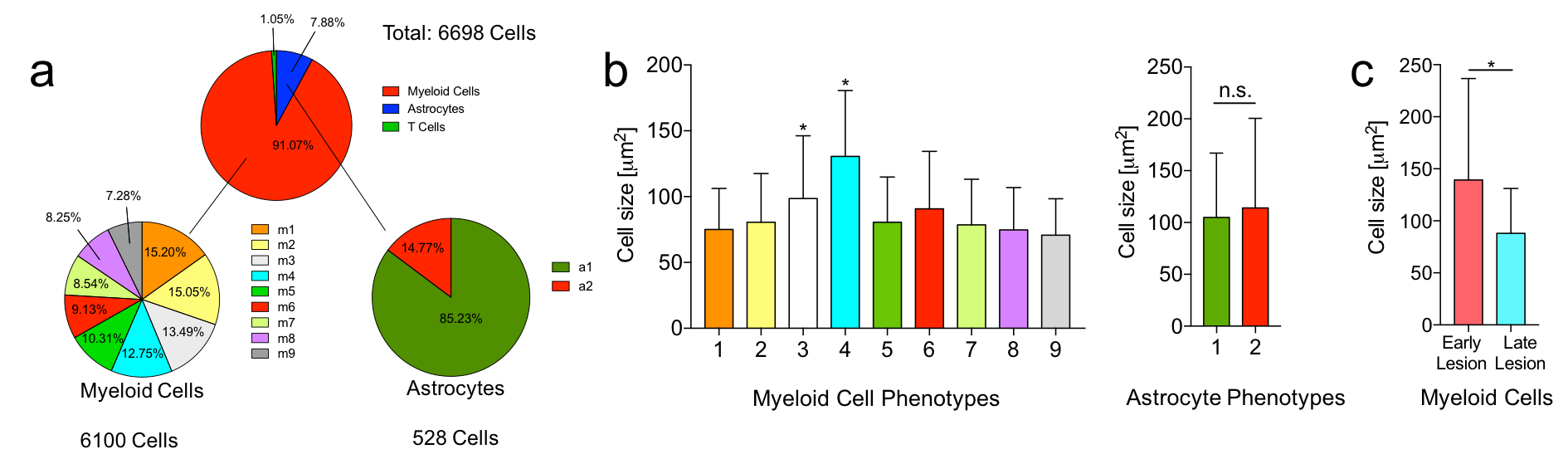
**

**Additional file 1: Figure S6** Late lesion cell phenotype frequency and size. **(a)** Relative quantities of each cell phenotype. **(b)** Cell phenotype size. Cells of myeloid phenotypes 3 and 4 (m3, m4), in the rim and perivascular space, respectively, are significantly larger than all other myeloid phenotypes, which occupy the lesion center. **(c)** Myeloid cell size per lesion. Myeloid cells in the early lesion are significantly larger than those in the late lesion. Data represent means + standard deviation. *p < 0.0001 by one-way ANOVA followed by the Tukey-Kramer multiple comparison test for comparison of myeloid cell phenotype sizes, and by unpaired Student’s t-tests for comparison of astrocyte phenotype sizes and lesion myeloid cell sizes. n.s. = not significant


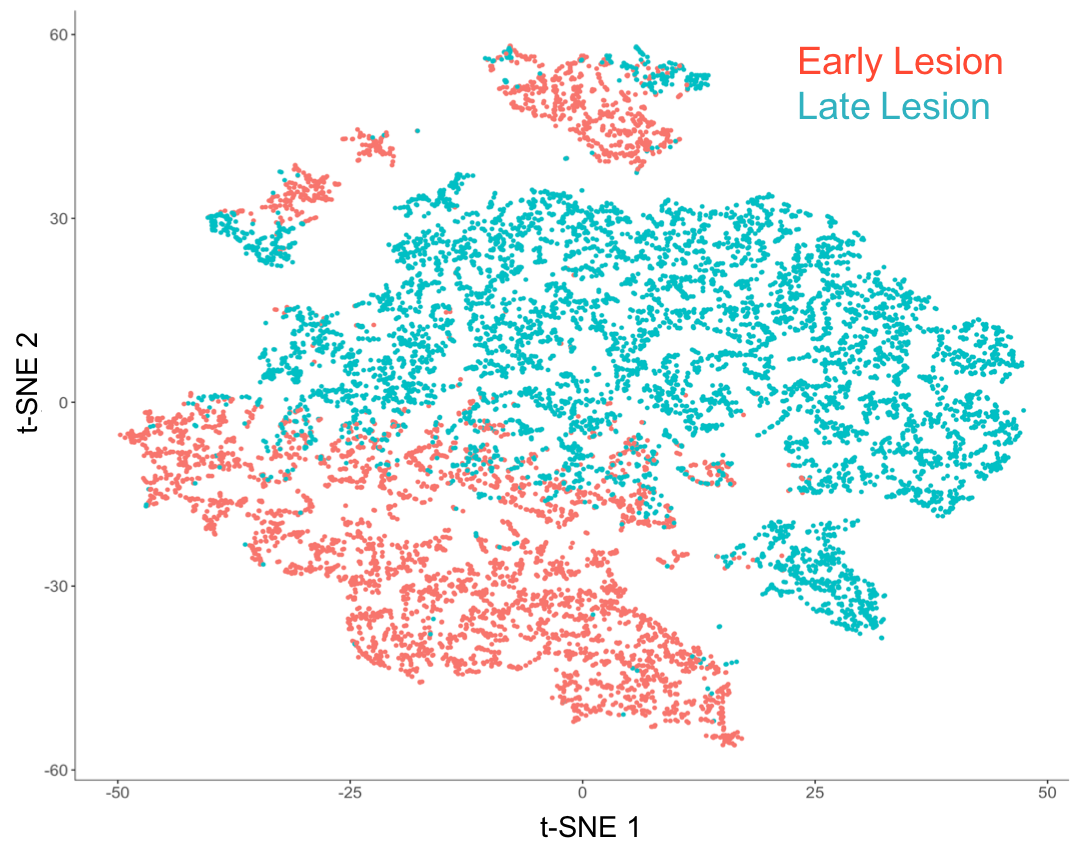


**Additional file 1: Figure S7** Comparison of lesion cell character with t-SNE. The plot shows all analyzed cells from the early and late lesions. Cellular populations overlap to some degree but are predominantly distinct

**
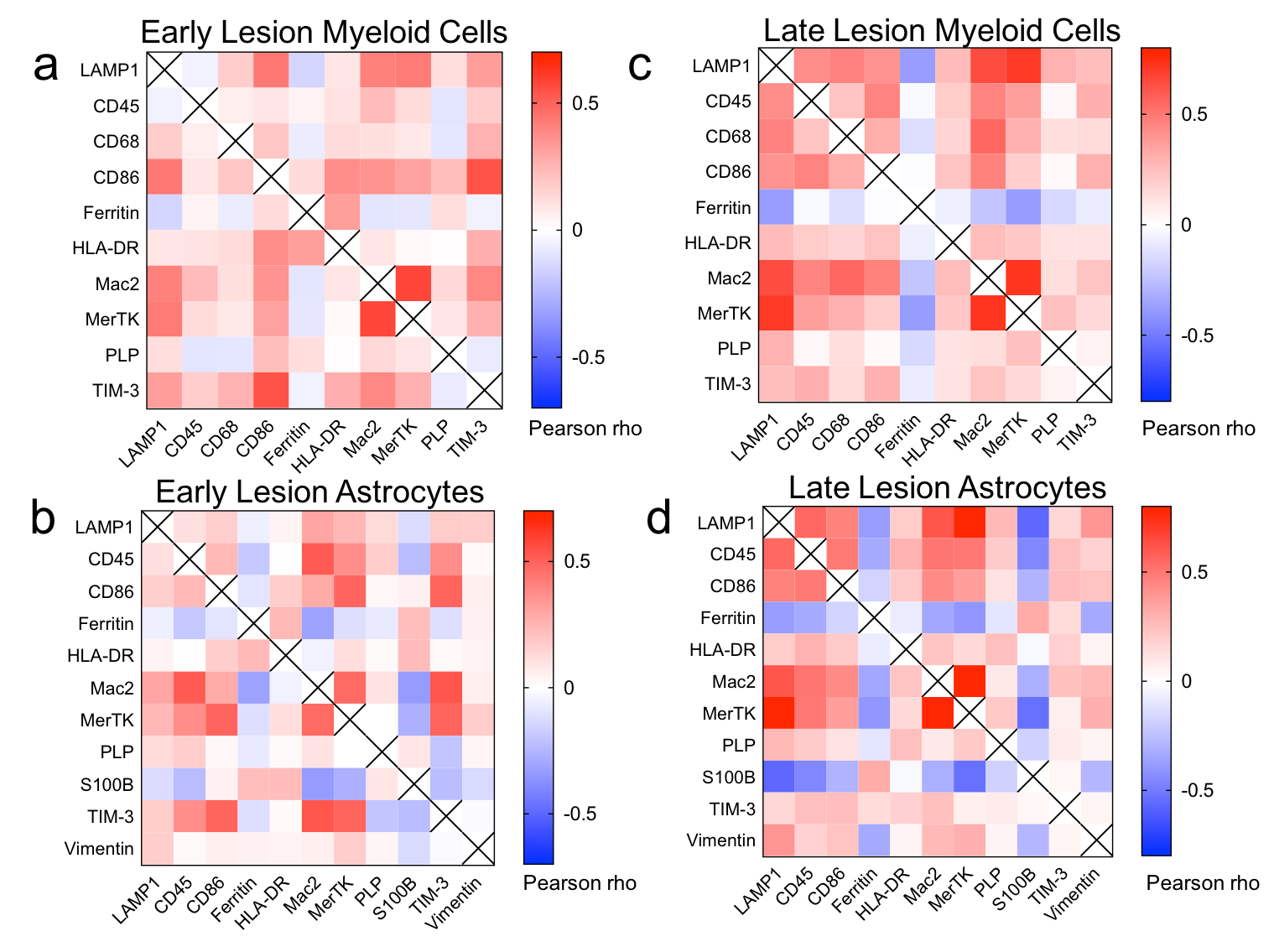
**

**Additional file 1: Figure S8** Pearson correlation matrices for myeloid cells and astrocytes. Matrices for early lesion **(a)** myeloid cells (n = 2913) and **(b)** astrocytes (n = 1431), and late lesion **(c)** myeloid cells (n = 6100) and **(d)** astrocytes (n = 528) are shown

**
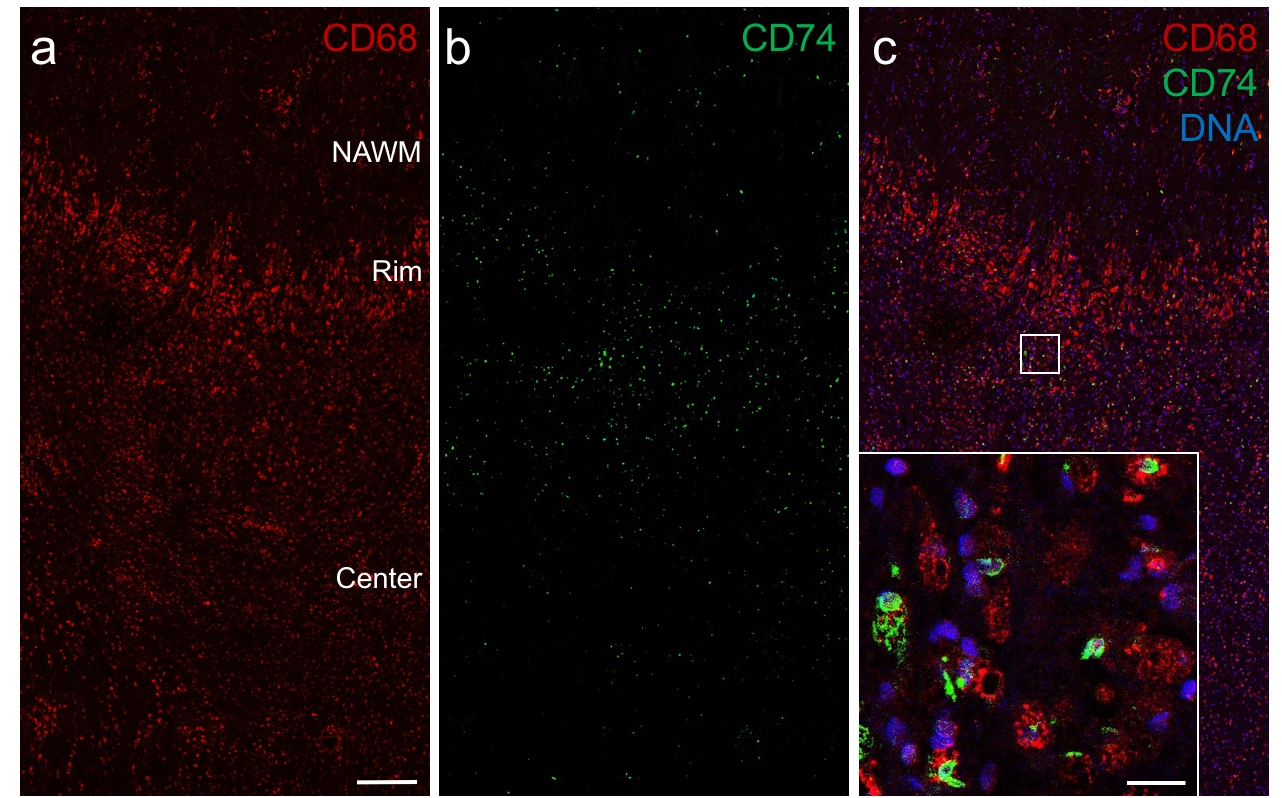
**

**Additional file 1: Figure S9** Immunofluorescence staining of CD74 in the rim of the early lesion. Images of **(a)** CD68 (red) and **(b)** CD74 (green) staining are shown separately and **(c)** merged with nuclear counterstaining (blue). The magnified inset in **(c)** corresponds to the area demarcated by the white square and shows expression of CD74 in CD68^+^ myeloid cells. Scale bars **a-c** = 200 μm. Inset scale bar in **c** = 20 μm. NAWM = normal-appearing white matter
